# Evidence of latent molecular diversity determining the virulence of community‐associated MRSA USA300 clones in mice

**DOI:** 10.1002/iid3.234

**Published:** 2018-08-08

**Authors:** Shiro Sonoda, Tetsuo Yamaguchi, Kotaro Aoki, Daisuke Ono, Ayami Sato, Chiaki Kajiwara, Soichiro Kimura, Yoshikiyo Akasaka, Yoshikazu Ishii, Yasunari Miyazaki, Naohiko Inase, Kazuhiro Tateda

**Affiliations:** ^1^ The Integrated Pulmonology Tokyo Medical and Dental University Tokyo 113‐8510 Japan; ^2^ Department of Microbiology and Infectious Disease Toho University, Faculty of Medicine Tokyo 143‐8540 Japan; ^3^ Department of Surgery Toho University Sakura Medical Center Chiba 285‐8741 Japan; ^4^ Department of Pathology Toho University. Faculty of Medicine Tokyo 143‐8540 Japan

**Keywords:** α‐Toxin, community‐associated MRSA, coagulase, Panton–Valentine leukocidin, USA300 clone

## Abstract

**Introduction:**

The USA300 clone of community‐associated MRSA is reported to be hypervirulent and epidemic in the United States. This clone causes a variety of diseases from lethal pneumonia to mild skin infections. We hypothesized that evolutionary diversity may exist among USA300 clones, which may link virulence traits with host responses and mortality rates.

**Methods:**

USA300 isolates from severe pneumonia (IP) and skin infection (IS) were characterized by pulsed‐field gel electrophoresis (PFGE) and next‐generation sequencing. Their virulence traits and host responses were compared in a lung infection model.

**Results:**

The two USA300 isolates were found to be identical in genomic analysis. Robust IL‐6 production, aggregation of bacteria, and hemorrhaging were observed in IP‐infected lungs, which were associated with a higher rate of mortality than that observed with strain IS. Few neutrophils were detected in the lungs infected with strain IP, even at high bacterial loads. Massive production of α‐toxin and coagulase were evident during the early phase of IP infection, and robust gene expression of *hla* (α‐toxin) and *lukS‐PV* (Panton–Valentine leukocidin), but not *coa*, *agrA*, or *rnaIII*, was confirmed in vitro. Strain IP also induced strong hemolysis in red blood cells.

**Conclusions:**

The present data demonstrated latent diversity in the virulence of USA300 clones. Unknown regulatory mechanisms, probably involving a host factor(s) as a trigger, may govern the virulence expression and resultant high mortality in certain sub‐clones of USA300.

## Introduction

Methicillin‐resistant *Staphylococcus aureus* (MRSA) was first reported in 1961 1. Over subsequent decades, hospital‐associated MRSA (HA‐MRSA), which remains of great concern as a cause of hospital infection in immunocompromised patients, has spread affecting hospitals and healthcare facilities across the world. In the 1990s, another threat became apparent, namely the appearance of community‐associated MRSA (CA‐MRSA) 2. CA‐MRSA exhibited hypervirulence and has been reported to circulate in communities through infections and/or colonization of healthy individuals, particularly children and young adults.

*S. aureus* strains are classified into 12 types from USA100 to USA1200 according to their banding patterns on pulsed‐field gel electrophoresis (PFGE) by the Centers for Disease Control and Prevention (CDC) 3. Among them, USA100 clone is a major HA‐MRSA clone, also known as New York/Japan clone, and USA300 clone is a major CA‐MRSA clone that is endemic in the USA and causes various types of infectious diseases, from skin and soft‐tissue infections to more invasive diseases, such as pneumonia, bacteremia, and osteomyelitis. Necrotizing pneumonia is a deadly form of USA300 infection, which is characterized by tissue necrosis, cavity formation, and resistance to antimicrobials.

USA300 clone has been reported to produce multiple virulence factors that are responsible for life‐threatening outcomes 4, 5, 6. Several toxins, including hemolysin (α‐toxin, β‐toxin, and γ‐toxin) and leukocidin (Panton–Valentine leukocidin [PVL], leukocidin D, and leukocidin E), are reported to play a crucial role in the pathogenesis of disease. These and other virulence factors cause host cell damage and tissue destruction via complex mechanisms. In particular, α‐toxin is widely accepted to exert cytotoxicity against a variety of cells and plays a major role in the pathogenesis of severe cases of pneumonia 7, 8, 9.

Expression of the multiple virulence factors of *S. aureus* is tightly regulated. Global regulatory systems, such as Agr and SaeRS, govern the expression of a range of virulence traits. There is limited information available for the effects and involvement of host‐derived factors and/or cells on virulence factor regulation at the site of infection.

In this study, we demonstrated the hypervirulence of USA300 clones isolated from a severe pneumonia case (IP) compared with that isolated from a mild skin disease case (IS) using a lung infection mouse model. We hypothesized that evolutionary diversity may exist among USA300 clones, which may link virulence traits with host responses and mortality rates. There are negative perceptions of using mouse models for examining the virulence of CA‐MRSA, in particular, it is known that PVL confers strong cytotoxic activity against neutrophils in humans and rabbits, but not mice 10. However, some studies have demonstrated the virulence of CA‐MRSA using infected mouse models 11, and in this study, the data demonstrated striking differences in the virulence of genetically identical IP and IS strains, characterized by toxin production, host responses, and mortality in a lung infection mouse model. As a result of the constant evolution of MRSA, it is likely that the diversity of USA300 clone is changing and expanding all the time, leading to the selection of more virulent sub‐clones in clinical settings.

## Results

### Molecular characterization of MRSA strains by pulsed‐field gel electrophoresis (PFGE) and whole genome sequencing (WGS)

Strains IP, IS, and BAA‐1556 showed the same PFGE banding pattern as USA300‐0114, which is a USA300 type strain on PFGE (Sup Fig. S1A). N315 strain is one of the New York/Japan clone belonging to USA100 clone, so it shows a completely different banding pattern to USA300‐0114. WGS analysis revealed that the predicted ORFs of IP and IS were 2,757 and 2,726 bp in length, respectively. Comparison between the IP and IS genome sequences with that of BAA‐1556 showed that 19 ORFs were present only in the IP genome and nine ORFs were present only in the IS genome, and the role of these strain‐specific ORFs was unknown. The sequences of the major virulence‐associated genes were the same between strains IP and IS, as shown in Sup Table S1. In addition, bacterial growth curves showed similar increases in turbidity in the four strains (IP, IS, BAA‐1556, USA300‐0114) (Sup Fig. S1B).

### Survival and body weight changes in mice infected with MRSA

**Figure 1:**
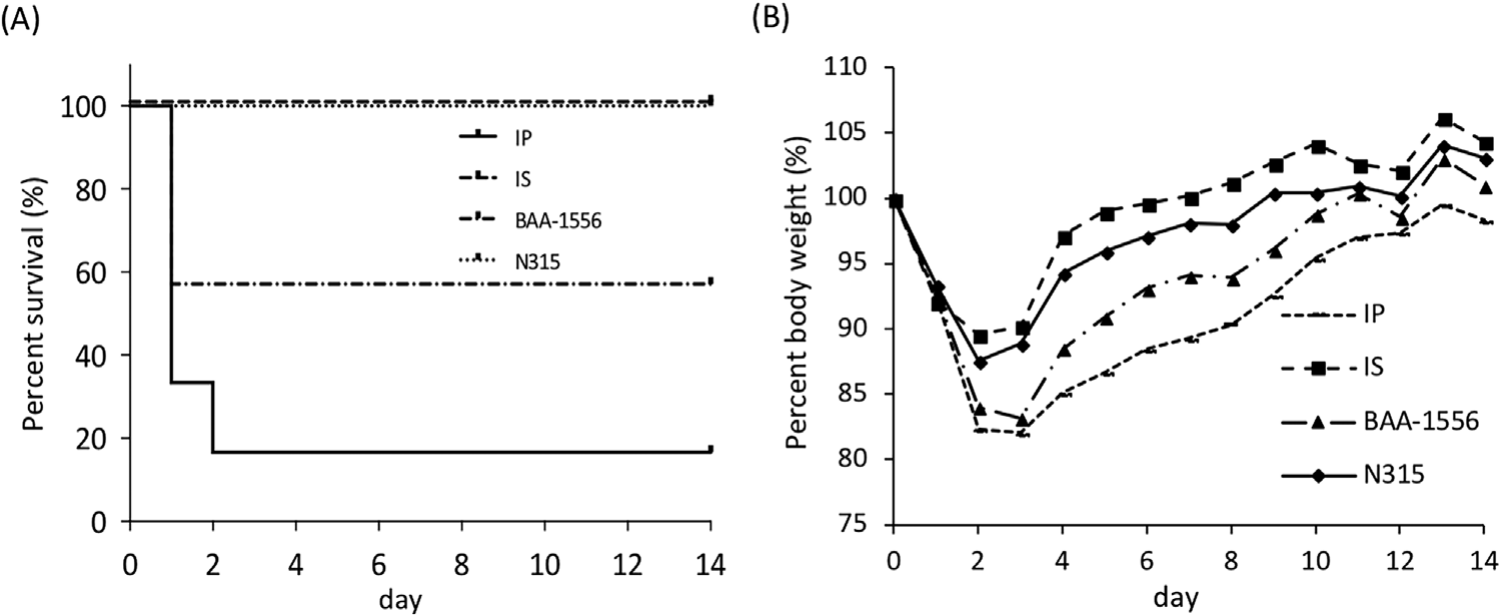
(A) Analysis of survival rates in the mouse pneumonia model. The survival rates for IP‐ and BAA‐infected mice were 16.7% and 33%, respectively, whereas IS‐ and N315‐infected mice showed 100% survival. The survival rates with strains IP and BAA were statistically significant compared with strain IS at *P* < 0.01. (B) Symbols: line, filled square, filled triangle, and filled diamond represent IP, IS, BAA and N315, respectively. Virulence was reflected by the loss of body weight of the infected mice. The greatest weight loss was seen in the IP group, followed by the BAA, N315, and IS groups. *IP versus IS *P* < 0.05; **BAA versus IS *P* < 0.05.

The mice were intra‐tracheally infected with several MRSA strains, including IP, IS, BAA‐1556, and N315, and then survival and body weights were monitored for 2 weeks. Mortality was observed in the mice infected with strains IP and BAA soon after infection. Approximately 80% and 40% mortality rates were recorded in IP‐ and BAA‐1556‐infected mice on Day 3, respectively, with no changes in survival being observed thereafter (Fig. 1A). By contrast, no deaths were observed in mice infected with strains IS or N315. In correlation with the survival data, apparent loss of body weight was higher in mice infected with strains IP and BAA, which showed more than 15% weight loss on Days 2 and 3 post‐infection (Fig. 1B).

### Cytokine production in the lungs of mice infected with MRSA

**Figure 2:**
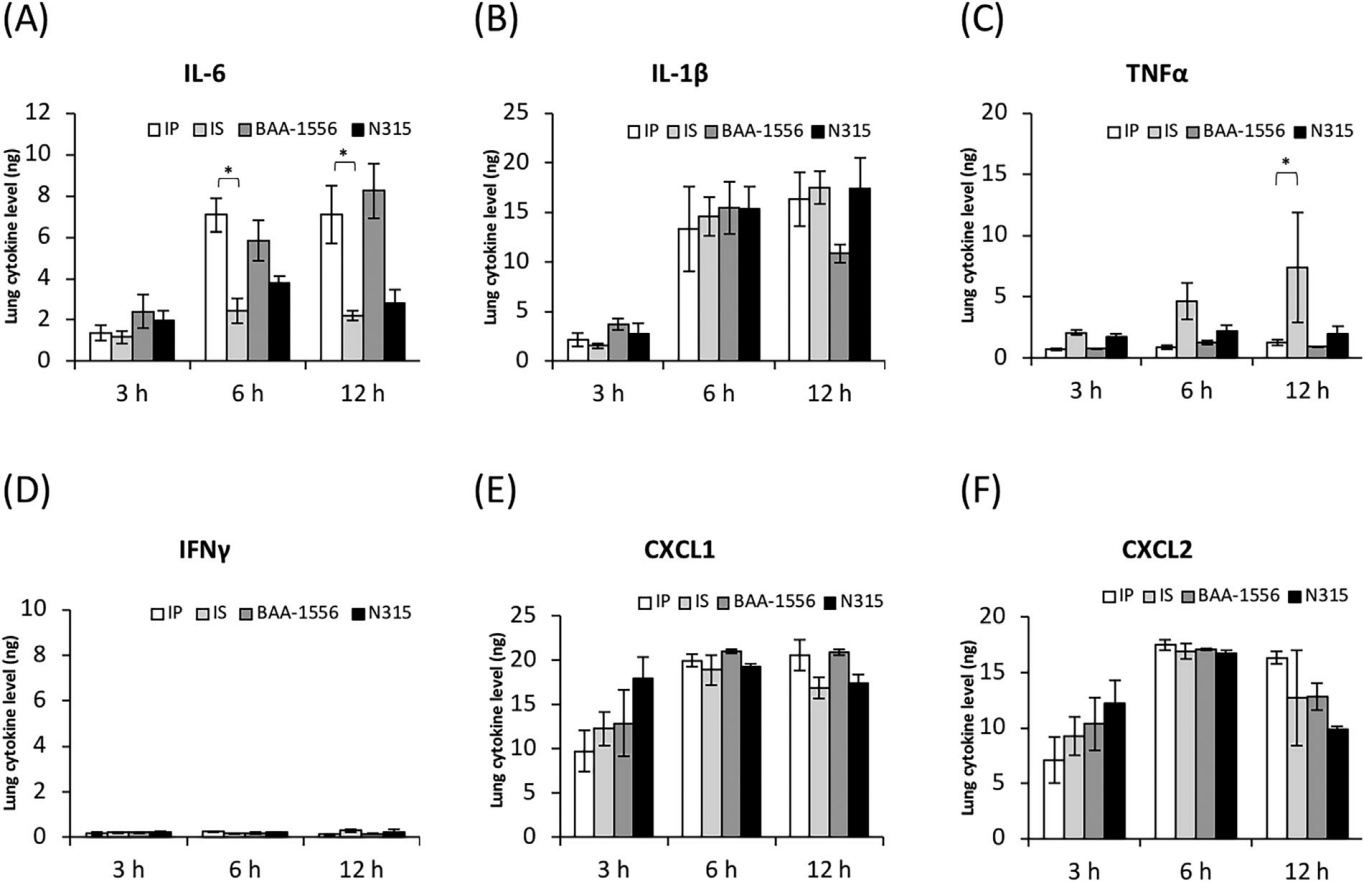
Expression levels of cytokines in the infected lungs at the designated times post‐infection. (A) IL‐6, (B) IL‐1β, (C) TNF‐α, (D) IFN‐γ, (E) CXCL1, and (F) CXCL2. *P < 0.0001 as determined by two‐way ANOVA.

To compare the early phases of host cytokine responses, production of IL‐6, IL‐1β, TNF‐α, IFN‐γ, CXCL1, and CXCL2 was examined 3, 6, and 12 h after infection with several MRSA strains (Fig. 2). Consistent with the survival data, relatively higher levels of IL‐6 were observed with strains IP and BAA‐1556 at 6 and 12 h, compared with those elicited by strains IS and N315. For TNF‐α, time‐dependent induction was observed for strain IS, but none of the other strains examined. There were no significant differences among these strains in terms of IL‐1β, CXCL1, and CXCL2 induction at these time points, and IFN‐γ was not detected under these experimental conditions.

### Histopathological characteristics of the lungs of mice infected with MRSA

**Figure 3:**
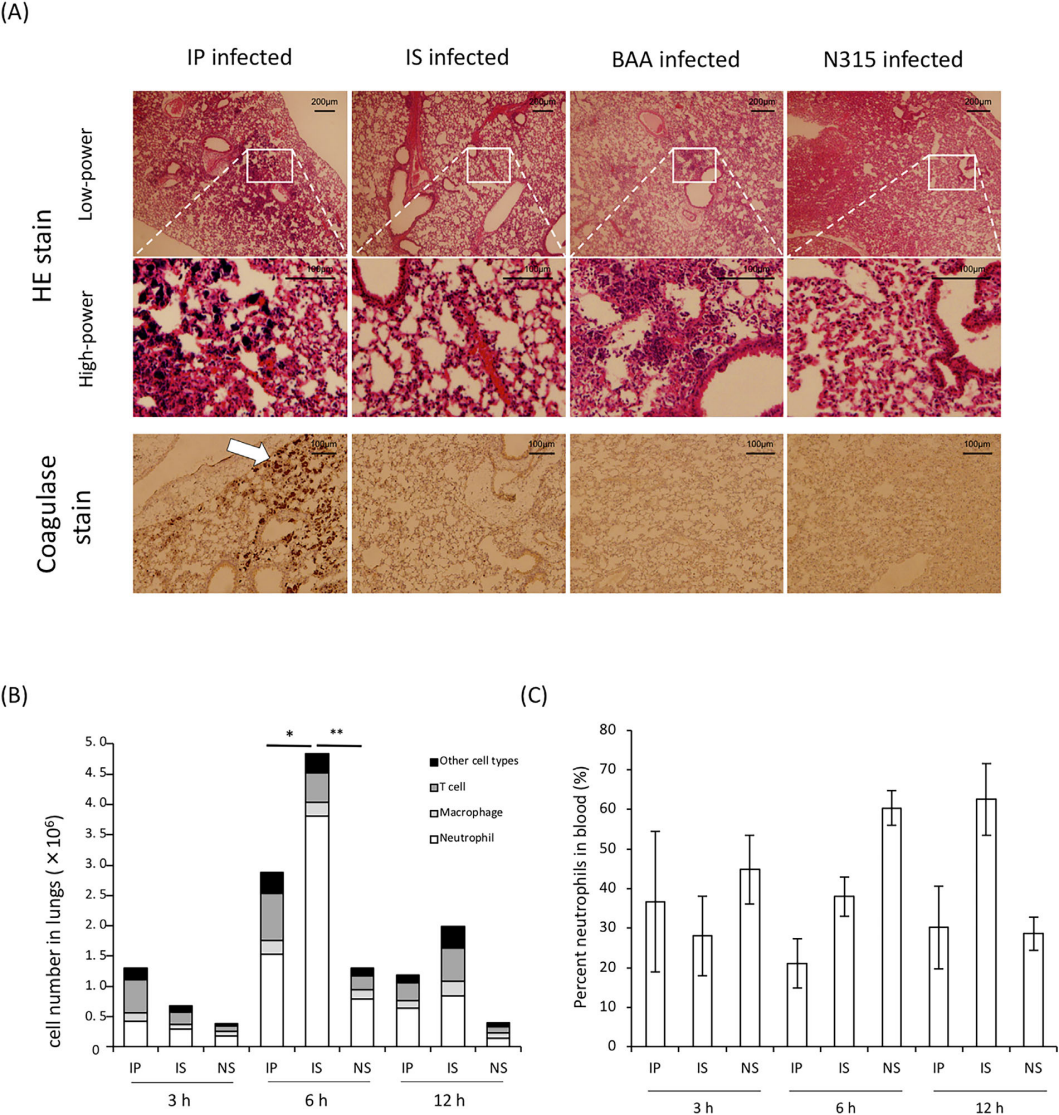
(A) Histopathology of lung tissues as determined by hematoxylin‐eosin staining. From left: IP‐, IS‐, BAA‐1556‐, N315‐infected lungs, respectively. Upper, low‐power field; middle, high‐power field. Scale bars indicate 200 μm and 100 μm in the low‐ and high‐power fields, respectively. IP‐infected lungs showed considerable bacterial aggregation and severe pneumonia compared with other USA300 clones. Lower, coagulase stained lung tissues. Scale bars indicate 100 μm. Arrows indicate staphylococcal aggregation and coagulase infiltration. (B) Numbers of immune cells in the lungs of mice and (C) percentage of neutrophils in the blood at the designated times post‐infection between clinical strains USA300, IP, and IS. Data are representative of two independent experiments, *n* = 3 per group. *Neutrophils of IP versus IS; *P* = 0.0001, **Neutrophils of IP versus NS; *P* < 0.0001 as determined by a *t*‐test.

Histopathologic analysis of the lungs of mice infected with MRSA at 12 h was performed using hematoxylin‐eosin stain (Fig. 3). In the lungs of mice infected with strain IP, considerable hemorrhaging accompanied by the accumulation of inflammatory cells and aggregated bacteria was demonstrated. At high magnification, numerous clustered cocci were observed in IP‐infected lungs, whereas no such bacterial agglutination was observed in the lungs of mice infected with strain IS. To a lesser extent, clustered bacteria were also evident in BAA‐1556‐infected lungs, but not in the lungs of mice infected with N315. Next, we examined coagulase production in the lungs by immune‐histochemical analysis (Fig. 3, coagulase stain). We confirmed production of coagulase in IP‐infected lungs (Fig. 3, IP‐infected coagulase stain); however, lower amounts of coagulase were evident in the lungs of IS‐challenged mice. The coagulase levels in the lungs of mice infected with strains BAA‐1556 and N315 were the same as those of IS‐infected mice.

### Changes in the bacterial burden and toxin production in the lungs of mice infected with MRSA

**Figure 4:**
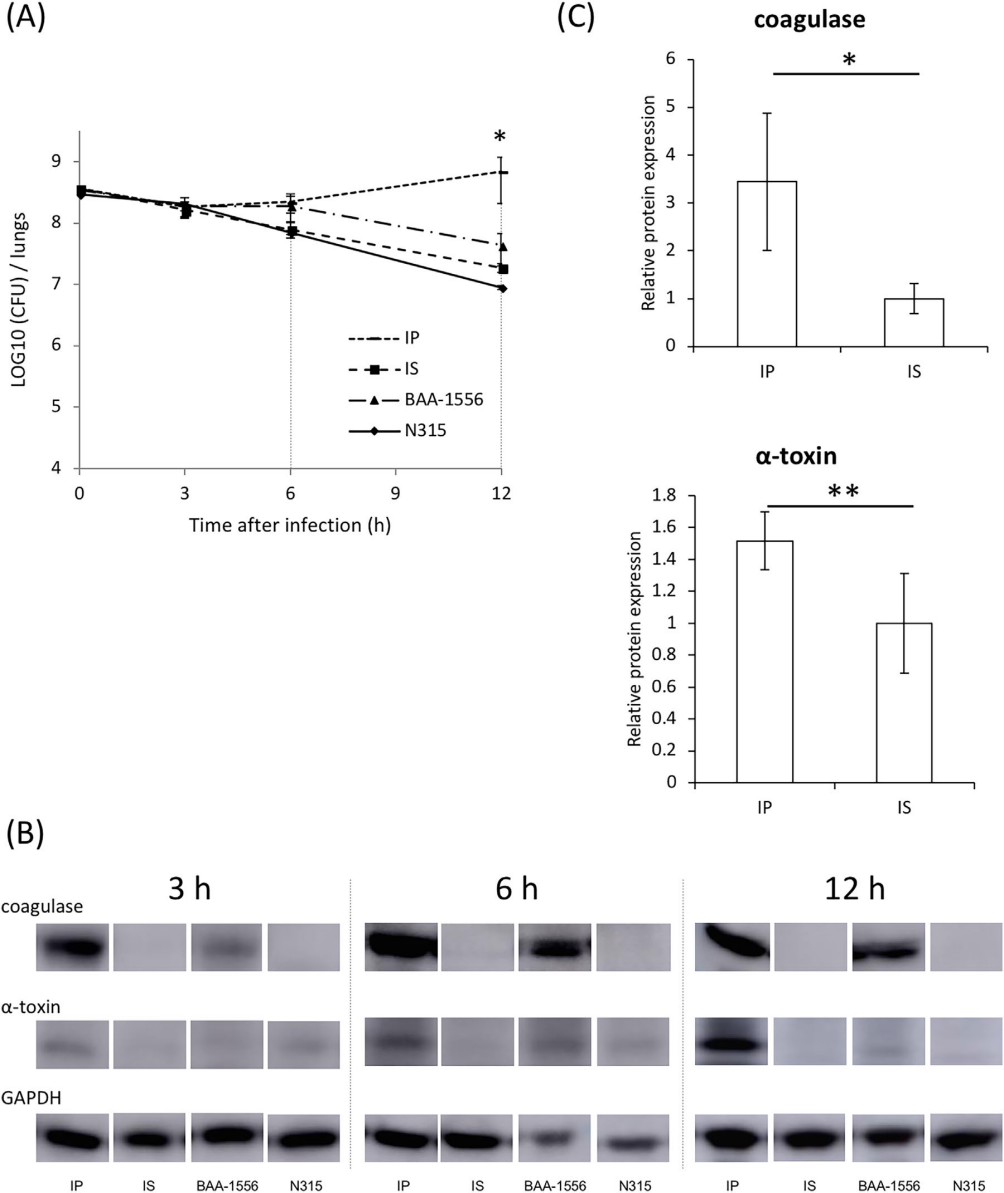
(A) Bacterial burden, as measured for both lungs together. At 3 h post‐infection, no differences in the bacterial burden in the lungs were evident between all four strain‐infected groups; however, at 12 h, a significantly higher bacterial burden was detected in the lungs of mice infected with IP compared with others, particularly IS‐infected mice at 12 hours post‐infection (7.0 ± 4.9 × 10^8^ vs. 1.9 ± 0.3 × 10^7^ CFU/lungs, respectively). *IP versus IS at 12 hours, *P* = 0.05 as determined by a *t*‐test. (B) Expression levels of α‐toxin and coagulase of the four strains were measured by western blotting. (C) Expression levels between clinical strains USA300, IP and IS, were measured by western blotting at 3 h post‐infection. GAPDH expression was analyzed as an endogenous control.

No differences in the bacterial burden in the lungs were evident between the IP‐ and IS‐infected groups at 3 h post‐infection; however, a significantly higher bacterial burden was observed in the lungs of mice infected with strain IP compared with IS‐infected mice at 12 h post‐infection (Fig. 4A). We next examined the production of coagulase and α‐toxin in the lungs of mice infected with MRSA. Of note, massive accumulation of coagulase was detected at 3 h in the lungs of mice infected with strain IP, but not those infected with strain IS, and these differences were evident at the later time points of 6 and 12 h. Regarding α‐toxin production, a weak band was visible at 3 h in the lungs of mice infected with IP, and the induction of α‐toxin was demonstrated in the lungs of IP‐infected mice, but not IS‐infected mice, at 12 h post‐infection (Figs. 4B and 4C).

### Differences in the host responses in the lungs and blood of mice infected with MRSA

Next, we examined the differences in host responses in the lungs and blood of mice infected with strains IP and IS. As shown in Figure 3B, significantly lower numbers of neutrophils were recorded in the lungs of IP‐infected mice compared with IS‐infected mice, although a higher bacterial burden was detected in the lungs infected with strain IP (Fig. 4A). There were no significant differences in the numbers of T cells and macrophages at all time points examined between the infections with strains IP and IS. Whereas, a lower percentage of neutrophils was detected in the peripheral blood of mice infected with strain IP compared with mice infected with strain IS (Fig. 3C).

### Hemolytic activity and virulence gene expression in strains IP and IS

*S. aureus* is known to possess a variety of cytotoxic and hemolytic toxins, such as α‐toxin and leukocidin. We examined the hemolytic activity of *S. aureus* after 3 h of growth in the presence of murine red blood cells. As shown in the samples after centrifugation (Sup Fig. S2A), macroscopic hemolysis was exhibited in the culture supernatant of strain IP, but not strain IS. To explore the factors contributing to this phenomenon, the transcription levels of several virulence genes were determined for strains IP and IS cultured with murine red blood cells. Among the factors examined, significantly higher *hla* and *lukS‐PV* transcription and expression were detected for strain IP than for strain IS, whereas no differences were observed for other factors, such as *agrA*, *rnaIII*, *clpA* (clumping factor), and *coa* (Sup Fig. S2B and C). These data suggested that *hla* and/or *lukS‐PV* may be responsible for the strong hemolysis induced by strain IP.

### Transcription and expression levels of virulence‐associated factors

**Figure 5:**
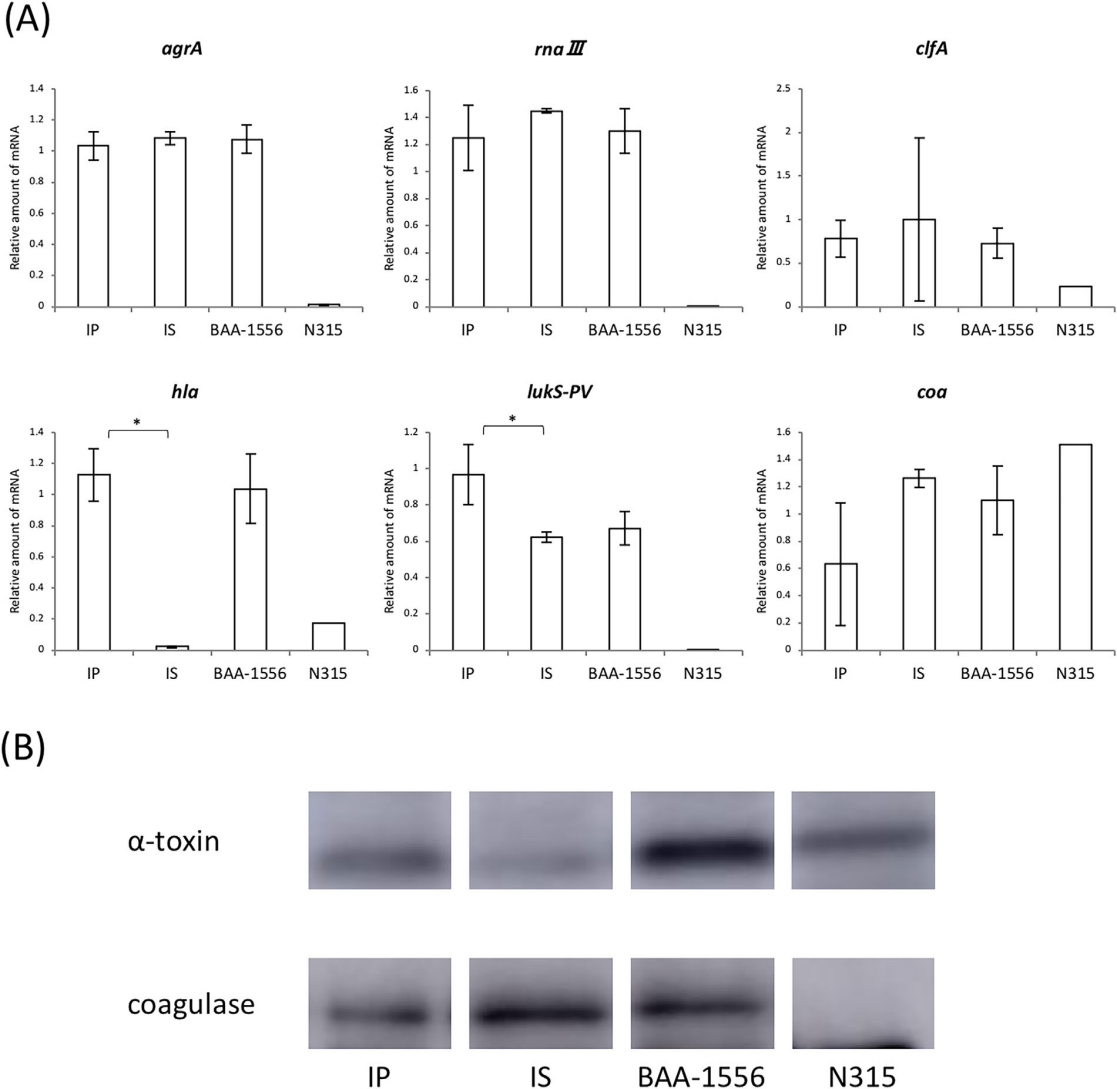
(A) Real‐time PCR of the broth supernatant after 12 h growth. *IP versus IS *P* < 0.001, **P < 0.05 as determined by a *t*‐test. (B) Western blotting after 8 h growth in BHI broth.

The transcription levels of representative regulatory factors were examined for strains IP and IS after incubation. Among the examined genes, no differences in expression were detected for *agrA*, *rnaIII*, or *clpA* between strains IP and IS. By contrast, significantly higher expression of the *hla* (encoding α‐toxin) and *lukS‐PV* genes was detected, along with lower expression of the *coa* gene in strain IP (Fig. 5A). Under these experimental conditions, the protein levels of α‐toxin correlated with the transcription data, with a stronger band being visible for strain IP by Western blot analysis (Fig. 5B). Compared with strain IS, higher production of α‐toxin was observed in strain BAA, which was similar to the results obtained with strain IP.

## Discussion

This study demonstrated the diversity in virulence of USA300 clones, by analyzing two genetically identical strains, IP and IS. Our findings shed new light on the latent ongoing molecular diversity in ubiquitously circulating USA300 clones throughout the world.

Strain IP was found to have significantly higher virulence, as determined by several host‐dependent parameters, including weight loss, IL‐6 levels, and survival. By contrast, higher TNF‐α levels were observed in IS‐infected lungs in a time‐dependent manner, although the reasons for this remain unknown. Histopathological data from IP‐infected lungs demonstrated the accumulation of inflammatory cells, hemorrhages, and the aggregation of bacteria, in addition to the massive deposition of coagulase. Importantly, neutrophil numbers were lower in IP‐infected mouse lungs at 6 h post‐infection compared with IS‐infected lungs, even when there was a higher bacterial burden. These data suggested that strain IP can cause massive inflammation, probably through the production of more virulence factors, such as cytotoxic toxins and aggregation‐associated proteins. Indeed, we found higher amounts of α‐toxin and coagulase in IP‐infected lungs than in IS‐infected lungs.

USA300 clones are known to be hypervirulent, although the exact molecular mechanisms mediating this virulence have not been clearly elucidated. It is tempting to speculate that the USA300 epidemic was driven by the acquisition of one or more “novel” virulence determinants, such as PVL. An alternative explanation for the hypervirulence of USA300 strains is that they have increased transcription of multiple core genomic global regulatory and downstream genes. Among the genes encoding virulence factors that are upregulated in USA300 are α‐toxin and PVL 12. The present study demonstrated higher gene expression of α‐toxin and PVL in strain IP than in strain IS. These data correlated well with the data for protein expression of toxins in the infected lungs. The higher levels of gene expression relating to toxin production may explain, at least in part, the pathological characteristics and mortality observed in IP‐infected mice.

The expression of multiple virulence factors, including α‐toxin and PVL, is controlled in a growth‐phase dependent manner by a number of global regulatory systems, including the *agr* and *sae* operons, each of which is also upregulated in USA300 isolates 12, 13, 14. For example, the expression of *agr* stimulates *rnaIII*, which results in the induction of α‐toxin 15 and PVL 16. Deletion of *saeR*, and to a lesser extent *agr*, resulted in attenuated expression of the genes encoding α‐toxin and PVL 14. In the present study, however, we observed no difference in the expression of *agrA*, *rnaIII*, and *saeR* between strains IP and IS.

Next‐generation sequencing analysis revealed conservation among 49 virulence factor genes, including the structural and regulatory sequences reported in the genomes of strains IP, IS, and BAA‐1556. In addition, promoter sequences located 100 bp upstream of the α‐toxin gene were identical between strains IP and IS. There were a few differences in the ORFs, although these were not functionally characterized and were annotated as hypothetical genes.

It is well known that mammalian hosts possess sensing mechanisms against invading pathogens (i.e., molecular pattern recognition) that when triggered lead to the induction of host defense systems. For bacteria, it is plausible to acquire the ability to sense, respond to, and evade host killing mechanisms, and bacteria including *S. aureus* are equipped with a number of evasion strategies. For example, it was reported that *saeR* was upregulated after phagocytosis of USA300 and USA400 strains by neutrophils 17. Bacteria may upregulate virulence factor expression, such as coagulase expression, in response to human defense systems. The molecular mechanisms that mediate the sensing of, and response to, host factors may be an interesting topic for future investigations.

There are several limitations in the present study. Although clear differences in virulence were seen between two genetically identical strains, we do not know whether this is a rare occurrence or common phenomenon in nature. We therefore need more investigations using large USA300 strain sets to examine infection site‐specific virulence differences. In addition, we could not identify the molecular mechanisms responsible for the higher virulence of strain IP in the lung infection model. Agr did not appear to be involved in this mechanism, and other regulatory factors therefore need to be investigated. Finally, most of these data were obtained using the mouse model of pneumonia. The host species may therefore be another variable, because PVL was reported to be active against human‐derived cells, but not equally active against other cell types. Future studies using human cells would therefore be of interest.

In conclusion, the present data demonstrated latent ongoing molecular diversity and evolution in USA300 clones. Further studies are now needed using novel techniques to differentiate between more and less virulent sub‐clones of USA300.

## Materials and Methods

### Animals

Specific‐pathogen‐free Balb/c mice were purchased from Charles River Laboratories (Kanagawa, Japan). Mice were maintained under specific‐pathogen‐free conditions within the animal care facility in the Laboratory Animal Research Center of Toho University School of Medicine until 6–8 weeks of age. Animal and pathogen protocols were approved by the Institutional Care and Use Committee (approval number 17‐55‐220, 17‐55‐58).

### Bacterial strains

We used two clinical MRSA strains that were isolated from cases of severe pneumonia (IP) and skin infection (IS) 18. As reference strains of MRSA, N315 (GenBank accession no. BA000018), USA300 clone ATCC BAA‐1556 (GenBank accession no. CP000255), and USA300‐0014 (JCSC6774) were also used. Each strain was cultured from frozen stocks onto BHI agar and incubated overnight at 35°C. Then, a single colony was inoculated into BHI broth and grown overnight at 35°C with shaking (160 rpm).

### Genetic analysis

PFGE typing was performed as previously described, using a contour‐clamped homogeneous electric field apparatus CHEF Mapper (Bio‐Rad, Tokyo, Japan) 3, 19, 20, 21. Running parameters were as follows: volts, 6 V/cm; temperature, 14°C; initial switch, 5.3 s; final switch, 34.9 s; time, 19.7 h; angle, 120°. Whole‐genome sequencing of IP and IS was carried out by next‐generation sequencing. DNA libraries were prepared with the Nextera XT DNA Preparation Kit and sequenced using a MiSeq sequencer (Illumina, San Diego, CA) in a 2 × 300 base pair paired‐end run. Obtained reads were trimmed of low‐quality sequences (the Phred quality score <Q30). The draft genome sequence data were assembled using CLC Genomics Workbench v 9.5.1 software (Qiagen, Aarhus, Denmark). To compare the genomic background of these strains, we checked whether the strains possessed the 49 known virulence factor genes (Sup Table S1) 22, 23, 24, 25, 26, 27, 28, 29. For gene prediction, we applied the prediction program MiGAP (http://www.migap.org/). The sequence data for strains IP and IS had been deposited in the DNA Data Bank of Japan (DDBJ) under accession numbers SAMD00106551 and SAMD00106552, respectively.

### Bacterial growth curves

For the analysis of growth curves, *S. aureus* was cultured overnight in 5 ml of BHI broth (final concentration: approximately 10^7^ CFU/ml) at 35°C with shaking. At the designated time points, the absorbance at 600 nm was measured using the Bio‐Spec mini (SHIMADZU, Kyoto, Japan).

### Mouse model of *S. aureus* lung infection

The overnight culture was washed by centrifugation and re‐suspended in saline to 1.0–3.0 × 10^10^ CFU/ml. Balb/c mice aged 6–8 weeks were anesthetized with a mixture of ketamine (12.5 mg/kg) and xylazine (15 mg/kg). Then, 30 μl (equal to 1.0–3.0 × 10^8^ CFU/mouse) of *S. aureus* suspension were administered via tracheal instillation. The actual numbers of infected bacteria were confirmed by plate counts. The survival rate and weight changes were monitored for 2 weeks. For the analysis of bacterial numbers and toxin production, mice were euthanized at the designated time points and the lungs were carefully removed for further examination.

### Detection of cytokines in the *S. aureus*‐infected lungs

The removed lungs were homogenized using a homogenizer (IKA, Osaka, Japan) in 1 ml of PBS with protease inhibitor. The homogenates were centrifuged at 5000*g* for 10 min at 4°C, and the supernatant was stored at −20°C. For quantification of IL‐6, IL‐1β, TNF‐α, IFN‐γ, CXCL‐1, and CXCL‐2, commercially available ELISAs were used (R&D Systems, Minneapolis, MN).

### Differentiation of cells in the infected lung tissues and blood

At the designed time points, small pieces of PBS‐washed lung tissue were incubated in RPMI 1640 media (Gibco, Tokyo, Japan) containing 2% FBS, 0.5 mg collagenase D ml^−1^ (Roche) and 150 µg DNase ml^−1^ (Roche, Tokyo, Japan) for 50 min at 37°C. To the blood samples, 10 μl of heparin was added, and the cells were washed with PBS. Cell suspensions from the lung and blood samples were passed through a strainer and centrifuged at 600*g* for 5 min. Pellets were washed with PBS, containing 0.5% BSA and 2 mM EDTA, and centrifuged. Then red blood cells were lysed using BD Pharm Lyse™ (BD Biosciences, Tokyo, Japan) according to the manufacturer's instructions. Cell suspensions with stain buffer (PBS with 2% BSA) were incubated with an anti‐Fc receptor‐blocking antibody (purified anti‐mouse CD16/32 antibody, clone 93) from BioLegend (San Diego, CA) for 15 min on ice to reduce nonspecific antibody binding. Cells were then washed with stain buffer and surface stained for 30 min on ice using each experimental design combination of FITC anti‐mouse Ly6G antibody, PerCP/Cy5.5 anti‐mouse CD11b antibody, PE/Cy7 anti‐mouse F4/80 antibody, PE‐CD45 anti‐mouse antibody from BioLegend, and APC‐CD3e anti‐mouse antibody from eBioscience Inc. (San Diego, CA). Isotype‐matched controls and single‐conjugate controls were always included. Cells were washed with stain buffer and fixed with 4% paraformaldehyde in PBS for 15 min, then washed and stored at 4°C prior to analysis by flow cytometry. Flow cytometry was performed on a FACSCanto II (BD Biosciences) and analyzed using FlowJo software (version 7.6.5; Tree Star, Ashland, OR). Controls were performed using cells stained with each labeled antibody individually.

### α‐Toxin and coagulase in the lungs infected with *S. aureus*

The extracts from murine lung homogenates at 3, 6, and 12 h after infection were examined by Western blotting. Primary antibodies were anti‐*Staphylococcus* alpha‐hemolysin antibody (Abcam, Tokyo, Japan) or anti‐coagulase antibody (Denka‐Seiken, Tokyo, Japan). The Amersham Imager 600 (GE imagination at work, Tokyo, Japan) was used for visualization 30. Then, we investigated in vitro the toxins present in the broth supernatants when the growth curve of MRSA was in stationary phase. Broth supernatants were centrifuged at 6,000g, and filtered using a EB‐DISK 25 0.45‐µm filter (Kanto Chemical, Tokyo, Japan).

### Histopathological analysis of the lungs infected with *S. aureus*

Lungs were removed 12 h after infection and were fixed in buffered 4% paraformaldehyde for histopathological examination. After embedding in paraffin wax, 6‐µm thick tissue sections were cut perpendicular to the anterior–posterior axis. The sections were placed on each polylysine‐treated slide and stained with hematoxylin and eosin, as reported 31. For the detection of coagulase in the lungs, immunostaining was performed, as previously described 31. Briefly, we used Histofine pH9 (Nichirei, Tokyo, Japan) for antigen activation, 3% H_2_O_2_ for blocking peroxidase, and serum‐free Protein Block (Dako, Tokyo, Japan) for blocking protein artifacts. The primary polyclonal antibodies used were anti‐coagulase antibodies (Denka‐Seiken) at a dilution of 1:100.

### Real‐time RT‐PCR

Each strain was cultured in BHI broth for 12 h after inoculation. The cultures were then pelleted by centrifugation (1,600*g*, 15 min, 4°C), and the bacterial RNA was protected using an RNAprotect Bacteria Reagent Kit (Qiagen, Tokyo, Japan), and isolated using the RNeasy Mini kit (Qiagen) as previously described 32. TURBO DNA‐free Kit (Thermo Fisher Scientific, Yokohama, Japan) was used to remove any contaminating DNA. Then, the RNA was reverse transcribed into cDNA using High‐Capacity cDNA Reverse Transcription Kits (Thermo Fisher Scientific) according to the manufacturer's instructions.

The PCRs for the ΔΔCT method were performed as follows: 95°C for 20 s, followed by 40 cycles of 30 s at 95°C and 30 s at 60°C. The 16S rRNA housekeeping gene served as an endogenous control to normalize the expressional levels between the samples. The DNA sequences of the primers are described in Sup Table S2 23, 24, 27, 33, 34, 35, 36, 37.

### Coagulase in the blood cultures

BHI broth was added to the blood from mice at 6.7% and then 1.3–1.7 ×10^10^ CFU of IP or IS was added. The cultures were shaken for 3 h, and then the broth supernatant was extracted before centrifugation and analyzed by Western blotting as described above.

### Statistical analysis

The data for survival rates, body weight changes, and other factors (cytokine, bacterial burden, regulators and toxins), which were collected from at least three independent experiments, were analyzed for significance using a Log‐rank test, two‐way ANOVA, and one‐way ANOVA, respectively. Statistical analysis was performed using the GraphPad Prism 6 software (MDF, Tokyo, Japan).

## Conflicts of Interest

All authors: No reported conflicts of interest. Conflicts that the editors consider relevant to the content of the manuscript have been disclosed.

## Supporting information

Additional supporting information may be found online in the Supporting Information section at the end of the article.

**Figure S1**. (A) PFGE of the SmaI‐digested strains.

**Figure S2**. (A) Macroscopic findings from the broth supernatants after 3 hours growth in BHI broth containing murine blood.

**Table S1**. (A) Major virulence‐associated genes of MRSA. Gene sequences of over 50 toxins and regulators were compared (all data not shown).

**Table S2**. PCR primers used in this study
