# Supplementary figures and images for: Evidence of latent molecular diversity determining the virulence of community‐associated MRSA USA300 clones in mice
Source: Immun Inflamm Dis. 2018 Aug 8;6(3):402–12. doi: 10.1002/iid3.234 (PMC6113770; doi:10.1002/iid3.234)

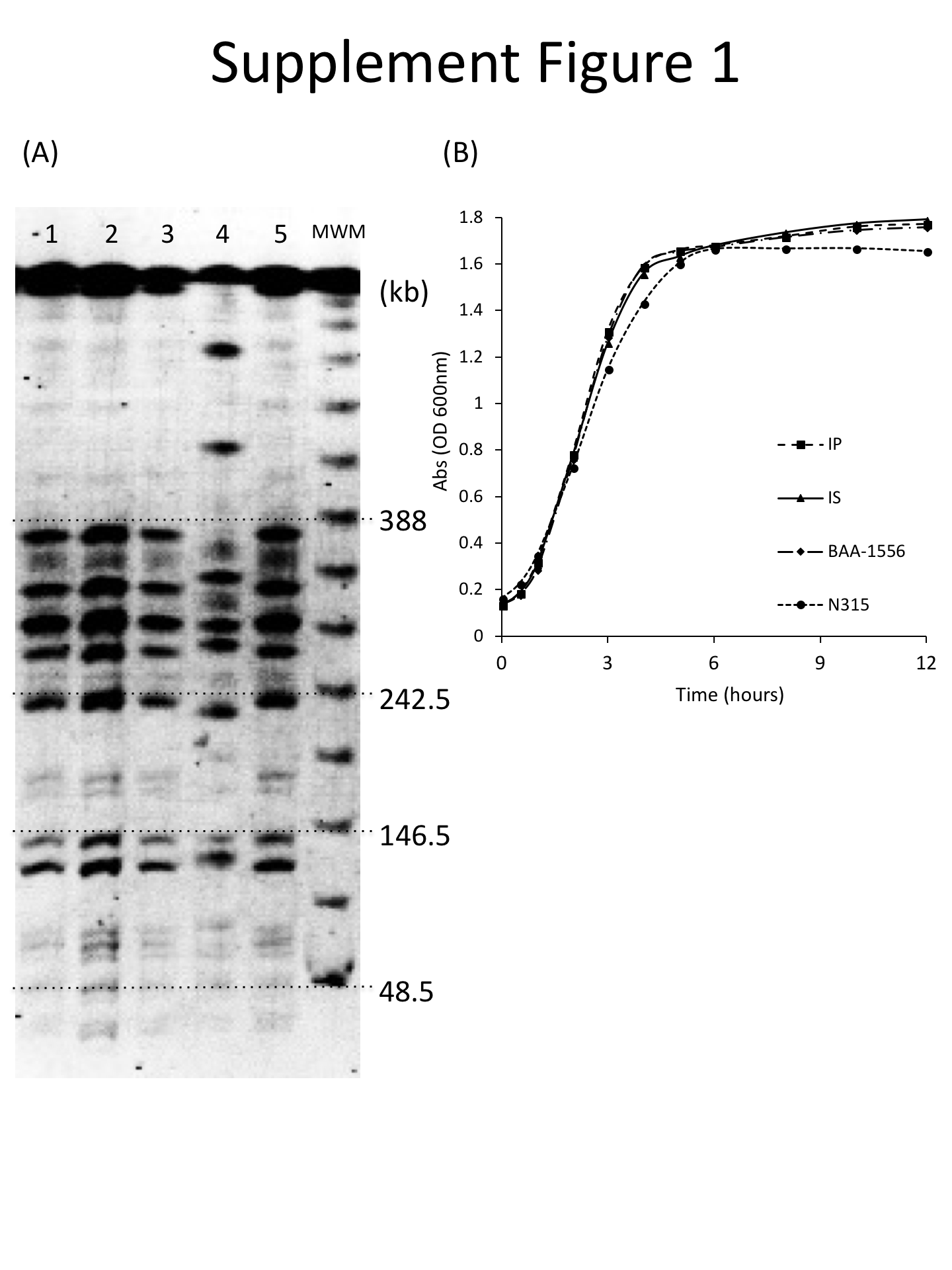

Supplement: Supplementary file 1 — Figure S1. (A) PFGE of the SmaI‐digested strains. [file IID3-6-402-s001.tiff]

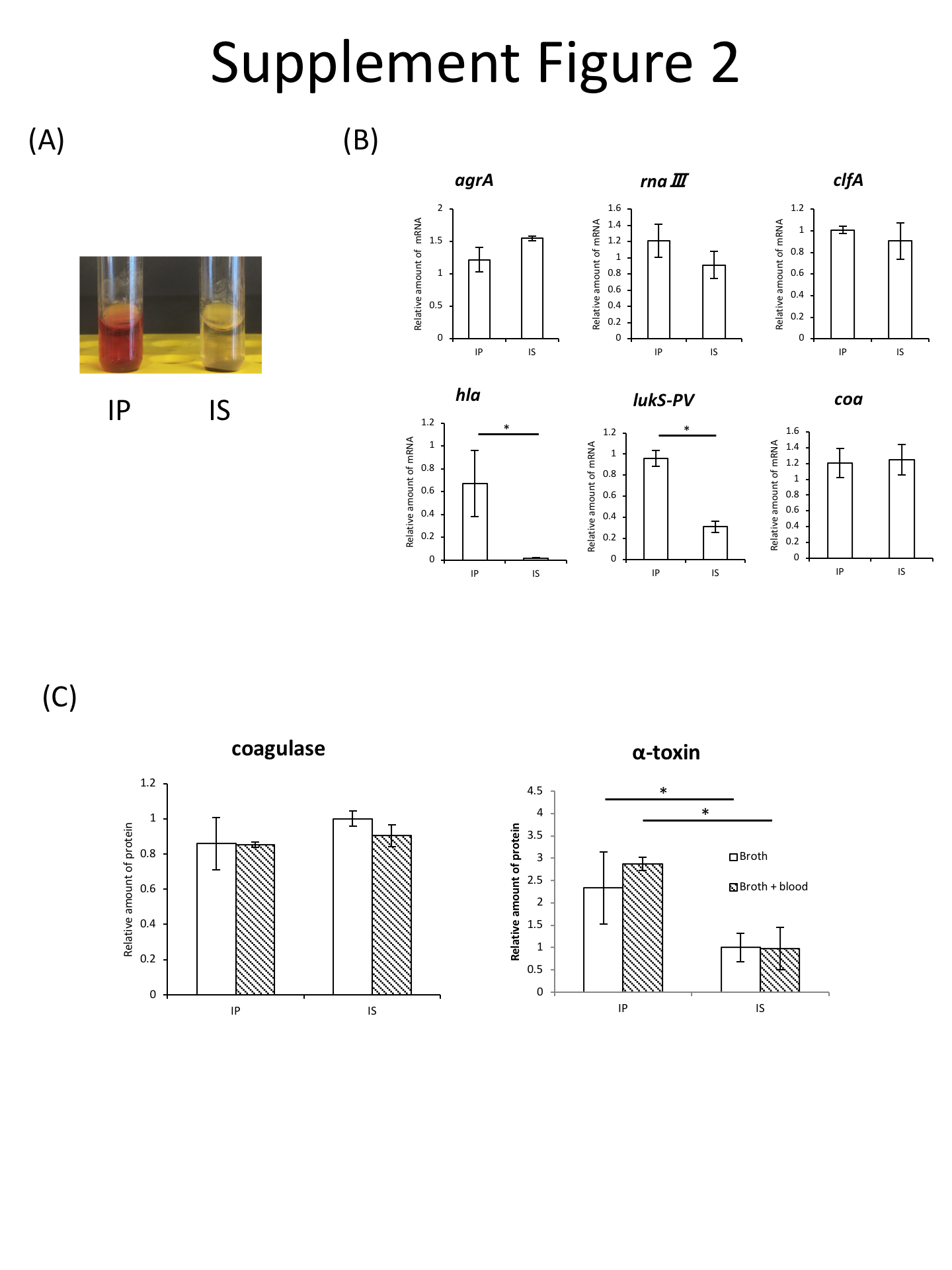

Supplement: Supplementary file 2 — Figure S2. (A) Macroscopic findings from the broth supernatants after 3 hours growth in BHI broth containing murine blood. [file IID3-6-402-s002.tiff]
